# Patterns and appropriateness of antipsychotic prescribing in community-dwelling older adults: a retrospective study

**DOI:** 10.3389/fphar.2026.1949586

**Published:** 2026-09-29

**Authors:** Khalid A. Alamer, Dana Alsugeir, Fawaz M. Alotaibi, Hussein Khalil, Mohammed J. Alfardan, Zahra A. Alrashed, Shahad S. Alghareeb, Mohammed M. Alsultan, Faten Alhomoud, Farah Kais Alhomoud, Yasser A. Alharbi, Bader R. Alzenaidy, Yousef Saeed Alqarni, Noha Keshk

**Affiliations:** 1 Department of Pharmacy Practice, College of Pharmacy, Imam Abdulrahman Bin Faisal University, Dammam, Saudi Arabia; 2 Pharmacy Practice Department, College of Pharmacy, Purdue University, West Lafayette, IN, United States; 3 Department of Pharmacy Practice, College of Pharmacy, Qassim University, Qassim, Saudi Arabia; 4 Saudi Food and Drug Authority, Riyadh, Saudi Arabia; 5 Department of Pharmacy Practice, Faculty of Pharmacy, Mansoura University, Mansoura, Egypt

**Keywords:** Aging, antipsychotic, geriatrics, medication safety, older adults, potentially inappropriate antipsychotic

## Abstract

**Background:**

Antipsychotics carry substantial risk in older adults, yet their use among community-dwelling outpatients remains insufficiently characterized in Saudi Arabia, where most prior work examined potentially inappropriate medications broadly or within psychiatric services. This study assessed patterns and appropriateness of antipsychotic prescribing and examined factors associated with prescribing without a documented qualifying indication among community-dwelling older adults across outpatient specialties.

**Methods:**

We conducted a retrospective cross-sectional study of patients aged 65 or older with a qualifying outpatient antipsychotic dispensing at King Fahad Hospital of the University, Saudi Arabia, from January 2019 to May 2023. The index date was the first qualifying antipsychotic dispensing. A criteria-based algorithm classified each patient’s prescribing appropriateness using FDA-approved indications, American Psychiatric Association guidelines, and the 2023 AGS Beers Criteria. Multivariable logistic regression identified factors associated with prescribing without a documented qualifying indication.

**Results:**

Among 394 patients, clearly appropriate prescribing was identified in 16.0%, while dementia- or delirium-related prescribing was classified as high-risk/context-dependent in 18.8%. Documented off-label prescribing was identified in 11.2%, 48.0% had no documented qualifying indication, and 6.1% had no diagnosis recorded within the ascertainment window. The latter group was excluded from comparative analyses. In the regression sample (n = 370), prescribing without a documented qualifying indication was more likely when the index antipsychotic was prescribed by medical or primary care (AOR 4.43, 95% CI 2.00 to 9.81; p < 0.001), neurology (AOR 2.42, 95% CI 1.27 to 4.63; p = 0.007), or other departments (AOR 3.16, 95% CI 1.16 to 8.63; p = 0.025) than by psychiatry.

**Conclusion:**

Prescribing without a documented qualifying indication was common outside psychiatry, but the absence of a qualifying diagnosis in the records does not confirm inappropriate prescribing. Systematic indication documentation, medication review, and targeted stewardship may improve prescribing quality in older adults.

## 1 Introduction

Older adults are at increased risk for medication-related harm due to the high prevalence of multimorbidity, polypharmacy, and age-related changes in pharmacodynamics and pharmacokinetics (Bartusik-Aebisher et al., 2026; Carlson and Yarns, 2023; McGettigan et al., 2024; Zazzara et al., 2021). These factors increase the likelihood of adverse drug events. Potentially inappropriate medications (PIMs) are those whose risks exceed the benefits, especially when safer or more effective alternatives exist (Spinewine et al., 2007; Choo, 2025). In older populations, PIM use has been linked to adverse outcomes such as falls, cognitive impairment, hospital admissions and readmissions, morbidity, and mortality (Moebs et al., 2020; Somers et al., 2010; Alsultan et al., 2025). Drug-related problems account for a substantial proportion of hospital admissions among older adults, with estimates ranging from 20% to 30% in some settings (Moebs et al., 2020; Somers et al., 2010). To address these concerns, the 2023 American Geriatrics Society Beers Criteria® provide a widely accepted framework for identifying medications that should generally be avoided, used with caution, or prescribed only under specific clinical circumstances in adults aged 65 years and older (2023 American Geriatrics Society Beers Criteria Update Expert Panel, 2023).

Antipsychotic medications represent a particularly important category of PIMs in older adults. Their use in this population is associated with adverse effects such as sedation, extrapyramidal symptoms, falls, cognitive decline, cerebrovascular events, and increased mortality (Seppala et al., 2019; Rogowska et al., 2023). Several antipsychotics also add to the anticholinergic burden, increasing the risk of confusion, drowsiness, falls, and functional decline among older individuals (Ozbilen et al., 2012; Grill et al., 2021). For the management of severe behavioral and psychological symptoms, their use carries significant risks, including falls, cerebrovascular events, cognitive deterioration, and increased mortality (Coles and Macfarlane, 2021; Hook et al., 2022). As a result, antipsychotic therapy in dementia should be tailored to the individual, prescribed for the shortest possible duration, and regularly reassessed with consideration for deprescribing when appropriate (Reus et al., 2016; Bjerre et al., 2018; Smith et al., 2025; Campbell et al., 2024; Abebe et al., 2021; Campbell et al., 2022).

Assessing the appropriateness of antipsychotic prescribing requires careful evaluation of both the medication and its clinical indication (Briesacher et al., 2005). Comprehensive documentation of the indication is critical to support assessment of prescribing quality and enable ongoing treatment review. However, documentation is often incomplete, especially when antipsychotics are prescribed outside psychiatric services and across various outpatient specialties where standardized medication review and deprescribing approaches may be lacking (Bärkås et al., 2023).

In Saudi Arabia, antipsychotic medications are prescribed to older adults, but there is limited evidence on the appropriateness of this practice (Al Odhayani et al., 2017; Alshehri et al., 2025). Most research in the region has addressed PIMs broadly, with few studies specifically examining antipsychotic prescribing (Alanazi et al., 2024; Alsaad et al., 2022; Jabri et al., 2023). A recent study among older adults with psychiatric disorders found potentially inappropriate antipsychotic use in 11% of patients, though it was limited to those attending psychiatric services (Alsultan et al., 2025). Therefore, the appropriateness of antipsychotic prescribing among community-dwelling older adults in broader outpatient settings remains insufficiently characterized. To address this gap, this study aims to evaluate the patterns and appropriateness of antipsychotic prescribing among community-dwelling older adults receiving outpatient care at a tertiary academic medical center in Saudi Arabia.

## 2 Methods

### 2.1 Study design and setting

This retrospective cross-sectional study evaluated the appropriateness of antipsychotic prescribing among community-dwelling older adults receiving outpatient care at King Fahad Hospital of the University (KFHU), a tertiary academic medical center in the Eastern Province of Saudi Arabia. Electronic health record data were extracted for the period from January 2019 to May 2023. The study was reported in accordance with the Strengthening the Reporting of Observational Studies in Epidemiology (STROBE) statement (von Elm et al., 2007).

### 2.2 Data sources

Patient-level data were obtained from two electronic health record datasets linked using the unique medical record number (MRN). The first consisted of outpatient pharmacy dispensing records containing demographic information, dispensing dates, prescribed antipsychotic agents, quantities dispensed, prescribing departments, and prescriber identifiers. The second comprised International Classification of Diseases, 10th Revision (ICD-10) diagnostic records including patient demographics, diagnosis codes, code descriptions, and diagnosis dates. Antipsychotic agents were identified using a predefined list of first- and second-generation agents developed from the World Health Organization Anatomical Therapeutic Chemical classification (World Health Organization, n.d.), Food and Drug Administration (FDA) labeling, (U.S. Food and Drug Administration, n.d.), and the KFHU institutional formulary. The complete list of antipsychotic agents used for medication identification is provided in the Supplementary Table S1.

### 2.3 Study population

Eligible patients were aged 65 years or older at the qualifying index date, defined as the first outpatient antipsychotic dispensing occurring at age 65 years or older. Patients without any outpatient antipsychotic dispensing at age 65 years or older were excluded. Because prescribing history before January 2019 was unavailable, patients whose qualifying index dispensing occurred near the start of the extraction period may have been prevalent rather than incident users. Therefore, the index date reflects the first qualifying dispensing observed in the study dataset rather than treatment initiation. Patient selection and inclusion in the analytic sample are summarized in Figure 1.

**FIGURE 1:**
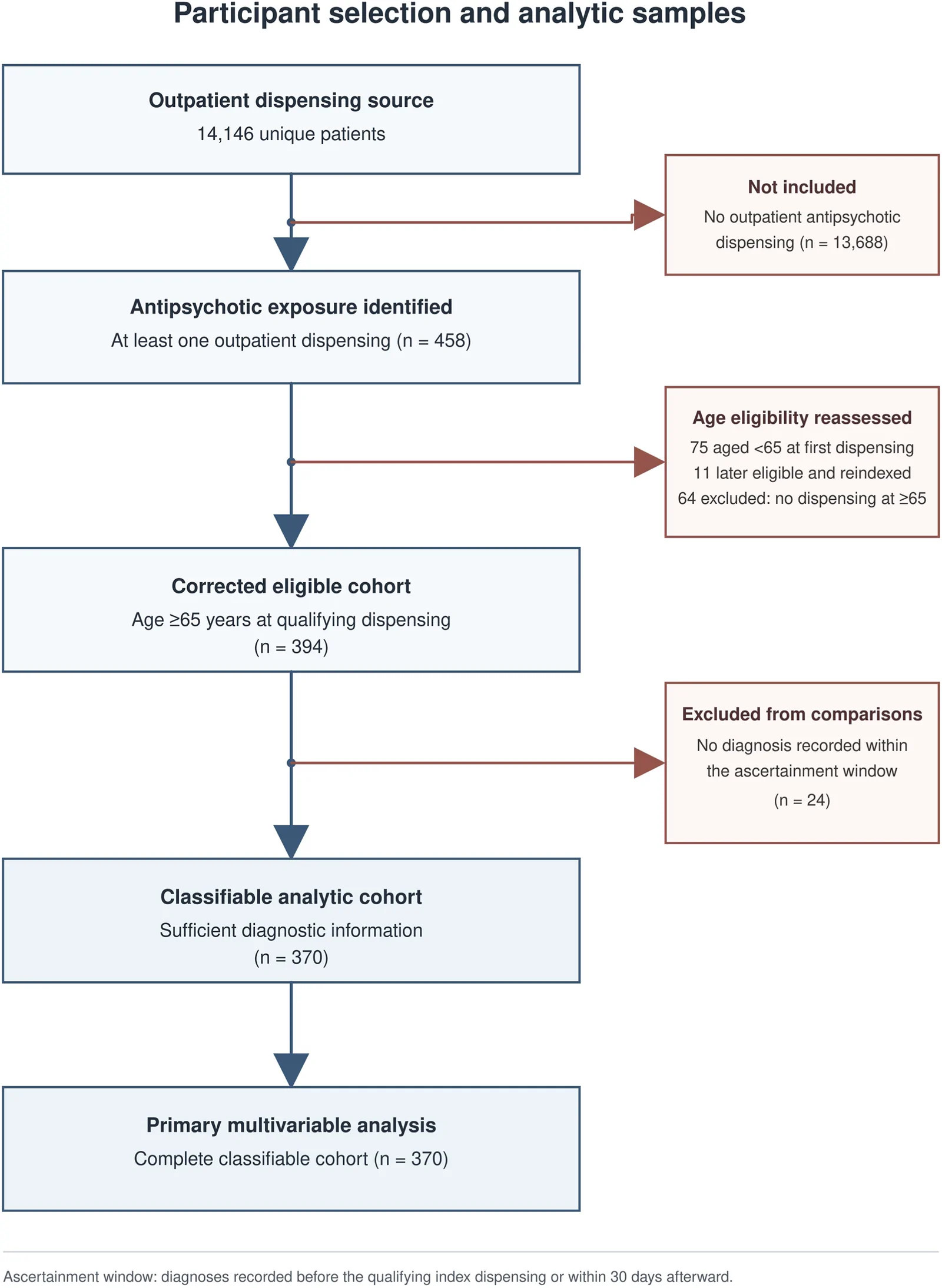
Flow of patients through cohort eligibility, classification, and primary analysis.

### 2.4 Assessment of prescribing appropriateness

A criteria-based classification algorithm was used to evaluate prescribing appropriateness, drawing on FDA-approved indications, American Psychiatric Association treatment guidelines, and the 2023 American Geriatrics Society Beers Criteria® (2023 American Geriatrics Society Beers Criteria Update Expert Panel, 2023; Reus et al., 2016; U.S. Food and Drug Administration). Classification was performed at the patient level according to diagnoses recorded within the prespecified ascertainment window. Patients were assigned hierarchically to one of five mutually exclusive categories. Clearly appropriate prescribing required a documented schizophrenia-spectrum disorder, bipolar-spectrum disorder, psychotic depression, or psychosis due to a medical or substance-related cause. High-risk/context-dependent prescribing included dementia or delirium. These patients were retained as a separate category and were not classified as clearly appropriate. Documented off-label prescribing included anxiety or trauma-related disorders, sleep disorder or insomnia, or depression without psychotic features. Prescribing without a documented qualifying indication was assigned when other diagnoses were present, but no diagnosis meeting the first three category definitions was identified within the ascertainment window. The no diagnosis recorded within the ascertainment window category was assigned when no diagnosis was recorded during that window. Parkinson disease without documented psychosis was not considered an antipsychotic indication. Prescribing an antipsychotic other than quetiapine or clozapine to a patient with Parkinsonism was retained as a drug-disease safety flag rather than an indication category. FDA-approved adjunctive treatment for major depressive disorder and short-term antiemetic use could not be confirmed because the available data did not capture concurrent antidepressant treatment, extended-release formulation, or antiemetic treatment intent. These exceptions were therefore not inferred from the antipsychotic medication alone.

Diagnoses were ascertained from ICD-10 codes recorded during any clinical encounter before the index dispensing date or within 30 days of the index dispensing date. The 30-day post-index window was included to account for documentation lag when the indication prompting treatment was recorded at a subsequent encounter within the same clinical episode. Diagnostic codes recorded after the index date were used for classification purposes only and do not establish temporal precedence of the indication. The complete ICD-10 code mapping and operational classification rules are provided in the Supplementary Material (Supplementary Table S2).

### 2.5 Study variables

Individual comorbidities were identified from ICD-10 diagnoses and included diabetes mellitus, hypertension, cerebrovascular disease, ischemic heart disease, heart failure, chronic kidney disease, dyslipidemia, and Parkinsonism. Parkinsonism was also evaluated as a drug-disease safety flag when the index antipsychotic was an agent other than quetiapine or clozapine. Additional variables included age at the index date, sex, marital status, prescribing department, and index antipsychotic agent. Prescribing departments and antipsychotic agents were grouped to reduce sparse-cell instability. Nationality and prescriber identifiers were not analyzed because of limited variability and incomplete data, respectively.

### 2.6 Statistical analysis

Patient characteristics were summarized using frequencies and percentages for categorical variables and means with standard deviations for continuous variables. Differences across the four classifiable prescribing categories were assessed using chi-square or Kruskal-Wallis tests, as appropriate. Patients with no diagnosis recorded within the ascertainment window were reported separately and excluded from comparative analyses. Diagnoses and clinical contexts contributing directly to category assignment were presented descriptively without statistical comparisons. Factors associated with prescribing without a documented qualifying indication were evaluated using multivariable binary logistic regression. Prescribing without a documented qualifying indication was coded as the outcome, while clearly appropriate, high-risk/context-dependent, and documented off-label prescribing were coded as the comparator. This comparator represented the presence of a documented indication or clinical context and was not interpreted as an appropriate-prescribing reference group. Prespecified predictors were age, sex, marital status, prescribing department, and index antipsychotic agent. Diagnostic variables used to define the classification categories were excluded to avoid circularity.

Two sensitivity analyses were conducted. The first compared all categories other than clearly appropriate prescribing with the clearly appropriate category. The second excluded the high-risk/context-dependent category and compared documented off-label prescribing and prescribing without a documented qualifying indication with clearly appropriate prescribing. Patients with no diagnosis recorded within the ascertainment window were excluded from all regression models. Analyses were performed using R version 4.6.1 (R Foundation for Statistical Computing, Vienna, Austria). All tests were two-sided, with p < 0.05 indicating statistical significance.

### 2.7 Ethical considerations

This study was conducted in accordance with the Declaration of Helsinki. Ethical approval was granted by the Institutional Review Board of Imam Abdulrahman Bin Faisal University, Dammam, Saudi Arabia (IRB-2026-05-0034, approved 14 January 2026). The requirement for individual informed consent was waived given the retrospective design and exclusive use of routinely collected, de-identified electronic health record data, with no direct patient contact or intervention.

## 3 Results

Of the 458 patients with at least one outpatient antipsychotic dispensing, 64 had no qualifying dispensing at age 65 years or older and were excluded. Eleven patients whose first observed dispensing occurred before age 65 years had a subsequent qualifying dispensing and were retained with their index date reassigned. The corrected cohort therefore included 394 community-dwelling older adults (Figure 1). Clearly appropriate prescribing was identified in 63 patients (16.0%), while 74 (18.8%) were classified as high-risk/context-dependent based on dementia or delirium. Documented off-label prescribing was identified in 44 patients (11.2%), and 189 (48.0%) had no documented qualifying indication. The remaining 24 patients (6.1%) had no diagnosis recorded within the ascertainment window and were reported separately and excluded from comparative analyses (Table 1). Patient characteristics differed across prescribing categories. The high-risk/context-dependent group was the oldest, with a mean age of 77.6 years (standard deviation [SD], 6.8), compared with 72.4 years (SD, 7.2) in the clearly appropriate group (p < 0.001). Women represented 64.1% of the classifiable cohort and were more prevalent in the high-risk/context-dependent (74.3%) and documented off-label (72.7%) groups, although the overall difference did not reach statistical significance (p = 0.054). None of the individual medical comorbidities differed significantly across the four classifiable groups. Psychiatry accounted for 68.6% of index prescriptions and predominated in the clearly appropriate (88.9%) and documented off-label (95.5%) groups. Neurology and medical or primary care departments contributed proportionally more prescriptions in the high-risk/context-dependent and no documented qualifying indication groups (p < 0.001) (Table 2). Schizophrenia-spectrum disorders (46.0%) and bipolar-spectrum disorders (42.9%) were the principal qualifying diagnoses in the clearly appropriate group, while psychosis due to a medical or substance-related cause was documented in 14.3%. Dementia was documented in 95.9% of the high-risk/context-dependent group, and delirium was documented in 4.1%. Among patients with a documented off-label context, anxiety or trauma-related disorders were most common (75.0%), followed by sleep disorder or insomnia (25.0%) and depression without psychotic features (2.3%). Five patients had Parkinsonism and received a nonpreferred index antipsychotic. Four had no documented qualifying indication, while one remained in the clearly appropriate group because a higher-priority qualifying indication was documented (Table 3). Among the 370 classifiable patients, quetiapine was the most frequently dispensed index antipsychotic (53.5%), followed by risperidone (20.3%) and olanzapine (15.1%). Quetiapine use was highest in the high-risk/context-dependent (66.2%) and documented off-label (65.9%) groups. Index antipsychotic selection differed across prescribing categories (p = 0.019) (Table 4). The multivariable analysis included 370 classifiable patients, of whom 189 had no documented qualifying indication. Compared with psychiatry, prescribing by a medical or primary care department was associated with greater odds of having no documented qualifying indication (adjusted odds ratio [AOR], 4.43; 95% confidence interval [CI], 2.00–9.81; p < 0.001). Higher odds were also observed for neurology (AOR, 2.42; 95% CI, 1.27–4.63; p = 0.007) and other departments (AOR, 3.16; 95% CI, 1.16–8.63; p = 0.025). Age, sex, marital status, and index antipsychotic agent were not independently associated with the primary outcome (Table 5).

**TABLE 1:** Antipsychotic prescribing classification among community-dwelling older adults aged ≥65 years (N = 394).

Prescribing category	n (%)	Category definition
**Clearly appropriate**	63 (16.0%)	At least one qualifying primary indication was documented: Schizophrenia spectrum disorder, bipolar spectrum disorder, major depression with psychotic features, or psychosis due to a medical or substance-related cause
**High-risk/Context-dependent**	74 (18.8%)	Dementia or delirium was documented without a higher-priority qualifying indication. These contexts were retained separately because antipsychotic use requires careful clinical justification and risk assessment in older adults
**Documented off-label context**	44 (11.2%)	Anxiety or trauma-related disorder, sleep disorder or insomnia, or depression without psychotic features was documented without a higher-priority qualifying indication
**No documented qualifying indication**	189 (48.0%)	At least one diagnosis was recorded within the ascertainment window, but none matched a predefined qualifying indication or clinical-context category
**No diagnosis recorded within ascertainment window**	24 (6.1%)	No diagnosis was recorded before the qualifying index dispensing or within 30 days afterward; these patients were reported separately and excluded from comparative analyses

Values are n (%). Percentages use the corrected eligible cohort (N = 394) as the denominator and may differ slightly from 100% because of rounding. Classification was hierarchical in the order shown. Complete code definitions and operational rules are provided in Supplementary Table S2.

**TABLE 2:** Demographic and clinical characteristics of community-dwelling older adults prescribed antipsychotics, stratified by prescribing classification (N = 394).

Characteristic	Clearly appropriate (n = 63)	High-risk/context-dependent (n = 74)	Documented off-label context (n = 44)	No documented qualifying indication (n = 189)	No diagnosis in window (n = 24)^a^	Overall classifiable (n = 370)	p-value^b^
*Demographics*							
Age at index dispensing, mean (SD)	72.4 (7.2)	77.6 (6.8)	72.4 (6.6)	75.2 (7.0)	84.3 (13.1)	74.9 (7.2)	**<0.001**
Female sex, n (%)	35 (55.6%)	55 (74.3%)	32 (72.7%)	115 (60.8%)	2/5 (40.0%)	237 (64.1%)	0.054
Married, n (%)	53 (84.1%)	56 (75.7%)	35 (79.5%)	148 (78.3%)	15 (62.5%)	292 (78.9%)	0.671
*Medical comorbidities*							
Diabetes mellitus, n (%)	11 (17.5%)	14 (18.9%)	10 (22.7%)	28 (14.8%)	NA	63 (17.0%)	0.601
Hypertension, n (%)	13 (20.6%)	11 (14.9%)	6 (13.6%)	16 (8.5%)	NA	46 (12.4%)	0.069
Cerebrovascular disease, n (%)	2 (3.2%)	7 (9.5%)	3 (6.8%)	22 (11.6%)	NA	34 (9.2%)	0.222
Ischemic heart disease, n (%)	4 (6.3%)	1 (1.4%)	2 (4.5%)	11 (5.8%)	NA	18 (4.9%)	0.448
Heart failure, n (%)	1 (1.6%)	2 (2.7%)	1 (2.3%)	4 (2.1%)	NA	8 (2.2%)	0.977
Chronic kidney disease, n (%)	1 (1.6%)	0 (0.0%)	1 (2.3%)	1 (0.5%)	NA	3 (0.8%)	0.488
Dyslipidemia, n (%)	7 (11.1%)	4 (5.4%)	3 (6.8%)	7 (3.7%)	NA	21 (5.7%)	0.174
Parkinsonism, n (%)	3 (4.8%)	3 (4.1%)	0 (0.0%)	8 (4.2%)	NA	14 (3.8%)	0.569
*Prescribing department*							
Psychiatry, n (%)	56 (88.9%)	47 (63.5%)	42 (95.5%)	109 (57.7%)	14 (58.3%)	254 (68.6%)	**<0.001**
Neurology, n (%)	3 (4.8%)	17 (23.0%)	0 (0.0%)	32 (16.9%)	4 (16.7%)	52 (14.1%)	NA
Medical/Primary care, n (%)	2 (3.2%)	6 (8.1%)	2 (4.5%)	34 (18.0%)	5 (20.8%)	44 (11.9%)	NA
Other, n (%)	2 (3.2%)	4 (5.4%)	0 (0.0%)	14 (7.4%)	1 (4.2%)	20 (5.4%)	NA

Values are n (%) unless otherwise indicated. SD, standard deviation; NA, not applicable because diagnostic information was unavailable or because the p-value applies to the overall variable.

^a^ Patients with no diagnosis recorded before the qualifying index dispensing or within 30 days afterward were shown separately and excluded from comparative analyses. Sex was available for 5 of these 24 patients; the percentage for female sex in this group uses the available-case denominator.

^b^ P-values compare the four classifiable groups (n = 370). Age was compared using the Kruskal-Wallis test; categorical variables were compared using Pearson’s chi-square test. Significant p-values are shown in bold.

**TABLE 3:** Documented diagnoses and clinical contexts underpinning antipsychotic prescribing classification among community-dwelling older adults aged ≥65 years (N = 394).

Characteristic	Clearly appropriate (n = 63)	High-risk/context-dependent (n = 74)	Documented off-label context (n = 44)	No documented qualifying indication (n = 189)	No diagnosis in window (n = 24)^a^	Overall classifiable (n = 370)
*Primary qualifying indications*						
Schizophrenia spectrum disorders, n (%)	29 (46.0%)	0 (0.0%)	0 (0.0%)	0 (0.0%)	NA	29 (7.8%)
Bipolar spectrum disorders, n (%)	27 (42.9%)	0 (0.0%)	0 (0.0%)	0 (0.0%)	NA	27 (7.3%)
Major depression with psychotic features, n (%)	0 (0.0%)	0 (0.0%)	0 (0.0%)	0 (0.0%)	NA	0 (0.0%)
Psychosis due to a medical or substance-related cause, n (%)	9 (14.3%)	0 (0.0%)	0 (0.0%)	0 (0.0%)	NA	9 (2.4%)
*High-risk/context-dependent diagnoses*						
Dementia, n (%)	4 (6.3%)	71 (95.9%)	0 (0.0%)	0 (0.0%)	NA	75 (20.3%)
Delirium, n (%)	1 (1.6%)	3 (4.1%)	0 (0.0%)	0 (0.0%)	NA	4 (1.1%)
*Documented off-label contexts*						
Anxiety or trauma-related disorders, n (%)	5 (7.9%)	1 (1.4%)	33 (75.0%)	0 (0.0%)	NA	39 (10.5%)
Sleep disorder or insomnia, n (%)	2 (3.2%)	3 (4.1%)	11 (25.0%)	0 (0.0%)	NA	16 (4.3%)
Depression without psychotic features, n (%)	0 (0.0%)	0 (0.0%)	1 (2.3%)	0 (0.0%)	NA	1 (0.3%)
*Safety flag*						
Parkinsonism with a nonpreferred index antipsychotic, n (%)	1 (1.6%)	0 (0.0%)	0 (0.0%)	4 (2.1%)	NA	5 (1.4%)

^a^Patients with no diagnosis recorded before the qualifying index dispensing or within 30 days afterward were shown separately and excluded from comparative analyses. Values are n (%) within each prescribing category. Diagnoses may overlap within categories; the final prescribing classification followed the hierarchy shown in Supplementary Table S2. Patients with no diagnosis recorded within the ascertainment window are shown separately as NA. No inferential tests were performed because the diagnosis variables contributed directly to the prescribing classification. Parkinsonism was evaluated as a safety flag rather than a qualifying indication. Parkinson disease without documented psychosis was not considered evidence of an appropriate antipsychotic indication.

**TABLE 4:** Index antipsychotic agent selection among community-dwelling older adults aged ≥65 years, stratified by prescribing classification (N = 394).

Characteristic	Clearly appropriate (n = 63)	High-risk/context-dependent (n = 74)	Documented off-label context (n = 44)	No documented qualifying indication (n = 189)	No diagnosis in window (n = 24)^a^	Overall classifiable (n = 370)	p-value^b^
*Index antipsychotic agent*							
Quetiapine, n (%)	26 (41.3%)	49 (66.2%)	29 (65.9%)	94 (49.7%)	12 (50.0%)	198 (53.5%)	**0.019**
Risperidone, n (%)	13 (20.6%)	14 (18.9%)	9 (20.5%)	39 (20.6%)	4 (16.7%)	75 (20.3%)	NA
Olanzapine, n (%)	11 (17.5%)	9 (12.2%)	2 (4.5%)	34 (18.0%)	6 (25.0%)	56 (15.1%)	NA
Aripiprazole, n (%)	4 (6.3%)	1 (1.4%)	3 (6.8%)	14 (7.4%)	0 (0.0%)	22 (5.9%)	NA
Haloperidol, n (%)	4 (6.3%)	0 (0.0%)	1 (2.3%)	5 (2.6%)	2 (8.3%)	10 (2.7%)	NA
Amisulpride, n (%)	1 (1.6%)	1 (1.4%)	0 (0.0%)	2 (1.1%)	0 (0.0%)	4 (1.1%)	NA
Clozapine, n (%)	3 (4.8%)	0 (0.0%)	0 (0.0%)	0 (0.0%)	0 (0.0%)	3 (0.8%)	NA
Paliperidone, n (%)	1 (1.6%)	0 (0.0%)	0 (0.0%)	0 (0.0%)	0 (0.0%)	1 (0.3%)	NA
Chlorpromazine, n (%)	0 (0.0%)	0 (0.0%)	0 (0.0%)	1 (0.5%)	0 (0.0%)	1 (0.3%)	NA

^a^Patients without a diagnosis recorded in the ascertainment window were shown separately and excluded from the test. ^b^The p-value compares the distribution of individual index antipsychotic agents across the four classifiable groups (n = 370) using Pearson's chi-square test. Values are n (%).

**TABLE 5:** Factors associated with no documented qualifying indication for antipsychotic prescribing among classifiable community-dwelling older adults: multivariable logistic regression (N = 370).

Predictor variable	AOR	95% CI	p-value
*Primary outcome: no documented qualifying indication (189 events)*			
*Prescribing department (reference: psychiatry)*			
Medical/primary care department	4.43	2.00–9.81	**<0.001**
Neurology department	2.42	1.27–4.63	**0.007**
Other departments	3.16	1.16–8.63	**0.025**
*Demographic characteristics*			
Age at index dispensing, per 1-year increase	1.00	0.97–1.03	0.831
Male sex (reference: female)	1.47	0.93–2.34	0.099
Not married/other (reference: married)	1.07	0.62–1.85	0.795
*Index antipsychotic agent (reference: quetiapine)*			
Olanzapine	1.26	0.65–2.46	0.490
Risperidone	1.30	0.74–2.28	0.353
Other agents	1.28	0.63–2.61	0.493

AOR = adjusted odds ratio; CI = confidence interval. Significant p-values are shown in bold.

The outcome compared patients with no documented qualifying indication (n = 189) with patients who had a documented primary indication, high-risk/context-dependent diagnosis, or documented off-label context (n = 181). The latter categories formed a documentation-based reference outcome and were not interpreted as equally appropriate. The model included age, sex, marital status, prescribing department, and index antipsychotic agent. Reference categories were female sex, married, psychiatry, and quetiapine.

Sensitivity analyses supported the primary department finding. When all classifications other than clearly appropriate prescribing were compared with the clearly appropriate category, prescribing by a medical or primary care department remained associated with greater odds of the outcome (AOR, 6.58; 95% CI, 1.46–29.62; p = 0.014). When the high-risk/context-dependent category was excluded, the association was also retained (AOR, 7.84; 95% CI, 1.73–35.63; p = 0.008). Full estimates from both sensitivity analyses are provided (Supplementary Table S3).

## 4 Discussion

This study evaluated the appropriateness of antipsychotic prescribing among community-dwelling older adults receiving outpatient care. Clearly appropriate prescribing was identified in 16.0% of patients. A further 18.8% were classified as high-risk/context-dependent based on dementia or delirium. Documented off-label prescribing accounted for 11.2%, while 48.0% had another diagnosis recorded but no documented qualifying indication. An additional 6.1% had no diagnosis recorded within the ascertainment window and were reported separately. By applying an indication-based classification across outpatient specialties, this study extends local evidence beyond broad PIM prevalence and psychiatric-service populations.

Antipsychotic prescribing in dementia and delirium warrants specific consideration because these diagnoses formed the high-risk/context-dependent category and were not combined with clearly appropriate indications. Antipsychotics are commonly used to manage the behavioral and psychological symptoms of dementia (BPSD), including psychosis, agitation, and sleep disturbance, which are difficult to manage (Kales et al., 2015; Mok et al., 2024). However, their use carries substantial risks, including cerebrovascular events, falls, and increased mortality (Rogowska et al., 2023; Tan et al., 2025). American Psychiatric Association guidelines recommend nonpharmacological strategies as first-line management and advise that antipsychotics be reserved for agitation or psychosis that is severe, initiated at the lowest effective dose, and continued for the shortest duration necessary (Reus et al., 2016). In practice, pharmacological treatment is often preferred, owing to limited provider training in behavioral approaches and the perception of faster symptom control (Kales et al., 2015; Haddad et al., 2014). Delirium accounted for only a small proportion of this category, but it was retained because antipsychotic use for delirium in older adults also requires careful assessment of clinical necessity, treatment duration, and potential harm (2023 American Geriatrics Society Beers Criteria Update Expert Panel, 2023).

Documented off-label prescribing accounted for 11.2% of patients and most commonly involved anxiety or trauma-related disorders and sleep disorder or insomnia. This documented off-label proportion was lower than the 22%–86% range reported in a systematic review of off-label antipsychotic prescribing in older adults (Carton et al., 2015). This comparison should be interpreted cautiously because our documented off-label category was limited to specified coded clinical contexts and was separated from the 48.0% with other diagnoses but no qualifying indication. Prescriptions in the latter group may represent undocumented off-label use, continued treatment for a diagnosis not captured in the available records, or another clinical rationale that could not be established from coded data.

One important finding was that 189 patients (48.0%) had at least one diagnosis recorded within the ascertainment window but no documented qualifying indication for antipsychotic treatment. This group was distinct from the 24 patients who had no diagnosis recorded within the ascertainment window. The absence of a qualifying diagnosis should not be interpreted as confirmation that prescribing was clinically inappropriate. Psychiatry accounted for 57.7% of prescriptions in the no documented qualifying indication group, indicating that this finding was not confined to nonpsychiatric departments. Possible explanations include incomplete coding, undocumented off-label use, or continued prescribing without regular review (McKean and Monasterio, 2012). Incomplete documentation of treatment indications in electronic health records is a recognized problem (Bärkås et al., 2023). Earlier studies found that 15%–40% of antipsychotic prescriptions lacked a documented psychiatric indication (Verdoux, 2025). Missing indication documentation complicates assessment of treatment risks and benefits, medication review, and deprescribing. Without a documented treatment goal, it is also difficult to determine whether continued exposure to metabolic, cerebrovascular, and mortality risks is justified (Bhardwaj et al., 2025; Chow et al., 2023). Compared with prior Saudi studies conducted in psychiatric populations, the present outpatient cohort captured prescribing across a broader range of clinical services. In an earlier Saudi study set in general adult psychiatric care, 94.3% of antipsychotic prescriptions were linked to a psychotic disorder (Alosaimi et al., 2016). Differences in patient age, clinical setting, data sources, and classification methods may partly explain why a larger proportion of patients in our cohort had no documented qualifying indication. Across outpatient specialties, antipsychotics may be prescribed or continued for behavioral symptoms, insomnia, agitation, or other clinical concerns without a qualifying diagnosis being captured in the available coded records.

Prescribing department was independently associated with the absence of a documented qualifying indication. Compared with psychiatry, the adjusted odds were more than fourfold higher for medical or primary care departments and were also significantly higher for neurology and other departments. These findings may reflect differences in indication coding, continuity of psychiatric diagnoses across services, prescribing context, and access to specialist review. They do not establish that prescriptions originating outside psychiatry were clinically inappropriate because valid indications may have been documented elsewhere or not captured in the available data. Nevertheless, the findings identify nonpsychiatric outpatient departments as important settings for medication review, indication documentation, and antipsychotic stewardship (2023 American Geriatrics Society Beers Criteria Update Expert Panel, 2023; Kerns et al., 2018). The separation of dementia and delirium from clearly appropriate prescribing was maintained in the sensitivity analyses. Prescribing by a medical or primary care department remained associated with the outcome when all classifications other than clearly appropriate prescribing were compared with the clearly appropriate category and when the high-risk/context-dependent category was excluded. This consistency indicates that the primary departmental association was not dependent on treating dementia or delirium as part of an appropriate-prescribing reference group. However, the wide confidence intervals in both sensitivity analyses warrant cautious interpretation. Age, sex, marital status, and index antipsychotic agent were not independently associated with the outcome. Quetiapine accounted for 53.5% of index prescriptions among classifiable patients and was most common in the high-risk/context-dependent and documented off-label groups. This is consistent with international reports of frequent off-label use for insomnia and anxiety (Carton et al., 2015). In Canada, Bakouni et al. reported that 78% of antipsychotic use among community-living older users was off-label, while a recent Finnish national study by Majuri et al. also documented extensive off-label use among community-dwelling older adults without Alzheimer’s disease (Bakouni et al., 2019; Majuri et al., 2026). Cross-study comparisons should remain cautious because the definitions of off-label use and the handling of dementia differed across studies. This pattern supports the need for targeted review of quetiapine prescribing in older adults, especially when used for sedation, sleep, or behavioral symptoms without a clearly documented indication.

This study has several strengths. Linking patient-level outpatient dispensing records with ICD-10 diagnoses allowed antipsychotic use to be evaluated against documented clinical indications rather than medication exposure alone. The revised hierarchy separated clearly appropriate prescribing, high-risk/context-dependent dementia or delirium, documented off-label contexts, prescribing without a documented qualifying indication, and the absence of diagnostic information within the ascertainment window. The complete code set and operational hierarchy are reported to support reproducibility. These categories have distinct implications for interpretation, medication review, and stewardship.

Several limitations should inform the interpretation of the findings. The index date represented the first qualifying dispensing observed during the extraction window, not necessarily treatment initiation. Because medication exposure before January 2019 was unavailable, some patients may have been prevalent users. The single-center setting may also limit generalizability to primary care and community settings with less specialist psychiatric input. In addition, diagnoses recorded within 30 days after the index dispensing were included, preventing confirmation that the documented indication preceded prescribing. Reliance on routinely coded ICD-10 data introduced the possibility of under-coding and missing documentation. Therefore, the absence of a documented qualifying indication does not confirm that no valid clinical indication existed. The available data also lacked symptom severity, previous nonpharmacological management, threat of harm, prescriber rationale, dose appropriateness, concurrent antidepressant treatment, extended-release formulation, short-term antiemetic intent, adherence, and clinical outcomes, including falls, cerebrovascular events, hospitalization, and mortality.

The findings support targeted antipsychotic stewardship for older adults. The higher likelihood of prescribing without a documented qualifying indication across medical or primary care, neurology, and other departments identifies nonpsychiatric outpatient services as priorities for indication documentation, medication review, and education on antipsychotic use in older adults and the AGS Beers Criteria (2023 American Geriatrics Society Beers Criteria Update Expert Panel, 2023). The frequent absence of a documented indication supports systematic recording of treatment indications to facilitate medication review and deprescribing (Campbell et al., 2024; Bärkås et al., 2023; Gupta et al., 2018; Goldberg et al., 2026). Practical measures include mandatory indication documentation, review prompts for older adults, defined reassessment intervals, and deprescribing pathways involving psychiatry, primary care, geriatrics, and pharmacy (Reus et al., 2016; Bjerre et al., 2018). Prospective multicenter studies incorporating clinical indications and treatment outcomes are needed to validate these findings and evaluate stewardship interventions.

## 5 Conclusion

Among community-dwelling older adults receiving outpatient care, 48.0% had no documented qualifying indication, 11.2% had a documented off-label context, and 18.8% had a high-risk/context-dependent diagnosis of dementia or delirium. The absence of a qualifying diagnosis in the available records does not confirm clinical inappropriateness but identifies prescriptions requiring further review. The odds of prescribing without a documented qualifying indication were higher when the index antipsychotic was prescribed by medical or primary care, neurology, or other departments rather than psychiatry. These results highlight the need to document the clinical indication for antipsychotic therapy at prescribing, especially for older adults managed across multiple outpatient specialties. To improve prescribing quality and ensure safer use in this vulnerable population, systematic medication reviews, regular reassessment of ongoing need, and targeted antipsychotic stewardship interventions are recommended. Prospective multicenter studies including clinical indication, symptom severity, and outcome data are needed to validate these findings and guide effective interventions.

## Data Availability

The original dataset is available upon request. Requests to access these datasets should be directed to KA, kaalamer@iau.edu.sa.
