# Supplementary material for: Patterns and appropriateness of antipsychotic prescribing in community-dwelling older adults: a retrospective study
Source: Front Pharmacol. 2026 Sep 29;17:1949586. doi: 10.3389/fphar.2026.1949586 (PMC13658605; doi:10.3389/fphar.2026.1949586)
Supplement: Supplementary file 1 [file Table1.docx]

**Supplementary Table 1. Antipsychotic Agents Included in the Medication Identification Algorithm**

| **Generation** | **Generic Agent** |
| --- | --- |
| First-generation | Chlorpromazine |
| First-generation | Fluphenazine |
| First-generation | Flupenthixol |
| First-generation | Haloperidol |
| First-generation | Perphenazine |
| First-generation | Thioridazine |
| First-generation | Trifluoperazine |
| First-generation | Zuclopenthixol |
| Second-generation | Amisulpride |
| Second-generation | Aripiprazole |
| Second-generation | Asenapine |
| Second-generation | Brexpiprazole |
| Second-generation | Cariprazine |
| Second-generation | Clozapine |
| Second-generation | Iloperidone |
| Second-generation | Lurasidone |
| Second-generation | Olanzapine |
| Second-generation | Paliperidone |
| Second-generation | Quetiapine |
| Second-generation | Risperidone |
| Second-generation | Ziprasidone |

Note: Agents were identified from outpatient dispensing records using generic medication names and the institutional formulary. Classification as first- or second-generation was used for medication identification and description; prescribing classification was determined from the documented clinical indication or context rather than antipsychotic generation.

**Supplementary Table 2. International Classification of Diseases, Tenth Revision Codes and Operational Rules Used in the Revised Prescribing Classification**

| **Domain** | **Hierarchy / Category** | **Condition** | **Exact ICD-10 Code(s) or Rule** | **Operational Use** |
| --- | --- | --- | --- | --- |
| Prescribing classification | 1. Clearly appropriate | Schizophrenia spectrum | F20, F21, F22, F23, F25, F28, F29 | Primary qualifying indication |
|  |  | Bipolar spectrum | F30, F31 | Primary qualifying indication |
|  |  | Major depression with psychotic features | F32.3, F33.3 | Primary qualifying indication |
|  |  | Psychosis due to a medical or substance-related cause | F06.0, F06.1, F06.2; F10.5, F11.5, F12.5, F13.5, F14.5, F15.5, F16.5, F17.5, F18.5, F19.5 | Primary qualifying indication |
|  | 2. High-risk / context-dependent | Dementia | F01, F02, F03, G30, G31.83 | Assigned after excluding category 1 |
|  |  | Delirium | F05 | Assigned after excluding category 1 |
|  | 3. Documented off-label context | Anxiety or trauma-related disorder | F41; F43.0, F43.1, F43.2 | Assigned after excluding categories 1-2 |
|  |  | Sleep disorder or insomnia | G47.0, G47.9 | Assigned after excluding categories 1-2 |
|  |  | Depression without psychotic features | F32.0, F32.1, F32.2, F33.0, F33.1 | Assigned after excluding categories 1-2 |
|  | 4. No documented qualifying indication | Other diagnosis present | At least one diagnosis in the ascertainment window and no category 1-3 code | Documentation-based primary outcome |
|  | 5. No diagnosis recorded within ascertainment window | No diagnosis available in window | No diagnosis dated on or before index or within 30 days after index | Reported separately; excluded from comparative analyses |
| Safety assessment | Not an indication category | Parkinsonism with nonpreferred index antipsychotic | G20 or G21 documented on or before index; index agent other than quetiapine or clozapine | Safety flag only; Parkinsonism without psychosis was not a qualifying indication |
| Descriptive comorbidities | Medical comorbidity | Diabetes mellitus | E10, E11, E12, E13, E14 | Descriptive analysis |
|  |  | Dyslipidemia | E78 | Descriptive analysis |
|  |  | Hypertension | I10, I11, I12, I13, I14, I15 | Descriptive analysis |
|  |  | Ischemic heart disease | I20, I21, I22, I23, I24, I25 | Descriptive analysis |
|  |  | Heart failure | I50 | Descriptive analysis |
|  |  | Cerebrovascular disease | I60, I61, I62, I63, I64, I65, I66, I67, I68, I69 | Descriptive analysis |
|  |  | Chronic kidney disease | N18 | Descriptive analysis |

Notes: ICD-10 = International Classification of Diseases, Tenth Revision. Listed codes included their subcodes unless a specific subcode was enumerated. Classification was hierarchical in the order shown. Diagnoses recorded before the qualifying index dispensing or within 30 days afterward were used for classification. Post-index diagnoses addressed documentation lag and did not establish temporal precedence.

F06.7-F06.9 were not classified as psychosis. Parkinsonism without a psychosis code was not treated as a qualifying indication. Depression without psychotic features was not classified as clearly appropriate because concurrent antidepressant treatment and, for quetiapine, the extended-release formulation could not be established from the available data. Short-term antiemetic intent could not be identified and was not inferred from the antipsychotic medication alone.

**Supplementary Table 3. Sensitivity Analyses of Factors Associated with
Antipsychotic Prescribing Classification**

| **Predictor Variable** | **AOR** | **95% CI** | **p-value** |
| --- | --- | --- | --- |
| ***Model A. Not Clearly Appropriate vs Clearly Appropriate (N=370; 307 Events)*** | | | |
| ***Prescribing Department (Reference: Psychiatry)*** | | | |
| Medical / Primary Care department | 6.58 | 1.46-29.62 | **0.014** |
| Neurology department | 3.25 | 0.94-11.15 | 0.061 |
| Other departments | 2.33 | 0.51-10.67 | 0.275 |
| ***Demographic Characteristics*** | | | |
| Age at index dispensing, per 1-year increase | 1.04 | 1.00-1.09 | 0.065 |
| Male sex (reference: female) | 0.75 | 0.42-1.36 | 0.350 |
| Not married / other (reference: married) | 1.18 | 0.54-2.60 | 0.677 |
| ***Index Antipsychotic Agent (Reference: Quetiapine)*** | | | |
| Olanzapine | 0.47 | 0.20-1.08 | 0.077 |
| Risperidone | 0.72 | 0.34-1.52 | 0.387 |
| Other agents | 0.36 | 0.16-0.81 | **0.014** |
| ***Model B. Documented Off-Label or No Qualifying Indication vs Clearly Appropriate, Excluding the High-Risk / Context-Dependent Category (N=296; 233 Events)*** | | | |
| ***Prescribing Department (Reference: Psychiatry)*** | | | |
| Medical / Primary Care department | 7.84 | 1.73-35.63 | **0.008** |
| Neurology department | 3.22 | 0.92-11.24 | 0.066 |
| Other departments | 2.59 | 0.55-12.24 | 0.229 |
| ***Demographic Characteristics*** | | | |
| Age at index dispensing, per 1-year increase | 1.03 | 0.98-1.07 | 0.220 |
| Male sex (reference: female) | 0.79 | 0.43-1.46 | 0.452 |
| Not married / other (reference: married) | 1.14 | 0.50-2.55 | 0.759 |
| ***Index Antipsychotic Agent (Reference: Quetiapine)*** | | | |
| Olanzapine | 0.45 | 0.19-1.08 | 0.075 |
| Risperidone | 0.75 | 0.35-1.63 | 0.470 |
| Other agents | 0.41 | 0.18-0.95 | **0.037** |

AOR = adjusted odds ratio; CI = confidence interval. Both models included age, sex, marital status, prescribing department, and index antipsychotic agent. Reference categories were female sex, married, psychiatry, and quetiapine. Significant p-values are shown in bold.
